# Differential Contribution of the Aryl-Hydrocarbon Receptor and Toll-Like Receptor Pathways to IL-8 Expression in Normal and Cystic Fibrosis Airway Epithelial Cells Exposed to *Pseudomonas aeruginosa*

**DOI:** 10.3389/fcell.2016.00148

**Published:** 2016-12-22

**Authors:** Lucie Roussel, Shantelle LaFayette, Dao Nguyen, Carolyn J. Baglole, Simon Rousseau

**Affiliations:** Meakins-Christie Laboratories, Department of Medicine, McGill University, McGill University Health Centre Research InstituteMontreal, QC, Canada

**Keywords:** TLR2, TLR5, inflammation, infection, epithelium, innate immunity, biofilm, cystic fibrosis

## Abstract

*Pseudomonas aeruginosa* are gram-negative bacteria that frequently infect the lungs of cystic fibrosis (CF) patients. This bacterium is highly responsive to changes in its environment, resulting in the expression of a diverse array of genes that may contribute to the host inflammatory response. *P. aeruginosa* is well-known to induce neutrophilic inflammation via the activation of Toll-Like Receptors (TLRs). Recently, it was shown that pyocyanin, a phenazine produced by *P. aeruginosa*, binds to the aryl hydrocarbon receptor (AhR), leading to neutrophilic inflammation as part of the host defense response. In this study, we have investigated the contribution of the TLR and AhR signaling pathways to the expression of the neutrophil chemoattractant IL-8 in response to *P. aeruginosa* diffusible material. Although both pathways are involved in IL-8 synthesis, the AhR played a greater role when planktonic *P. aeruginosa* was grown in a media favoring phenazine synthesis. However, when *P. aeruginosa* was grown in a media that mimics the nutritional composition of CF sputa, both pathways contributed similarly to IL-8 synthesis. Finally, when *P. aeruginosa* was grown as a biofilm, the TLR pathway did not contribute to biofilm-driven IL-8 synthesis and AhR was found to only partially contribute to IL-8 synthesis, suggesting the contribution of another unknown signaling pathway. Therefore, the interaction between *P. aeruginosa* and airway epithelial cells is very dynamic, and sensor engagement is variable according to the adaptation of *P. aeruginosa* to the CF lung environment.

## Introduction

A complex relationship exists between the airway epithelium and potential airborne pathogens it encounters. Detection and discrimination of inhaled compounds and microorganisms require different sets of sensors to mount an appropriate response, which in presence of pathogens or toxicants, involves triggering inflammation. *Pseudomonas aeruginosa* is an opportunistic pathogen that frequently infects the airways of cystic fibrosis (CF) patients (Lipuma, 2010). Bacterial infection in CF lung disease is associated with neutrophilic inflammation that, over the course of the disease, leads to lung tissue damage and progressive loss of pulmonary function (Cantin et al., 2015).

The initiation of innate immunity through activation of airway epithelial sensors is a key contributor to this process (Wu et al., 2005). Toll-like Receptors (TLRs) are a well-characterized sensor family implicated in the detection of diverse microorganisms (Kawai and Akira, 2010). Bronchial airway epithelial cells recognize *P. aeruginosa* through binding of flagellin to TLR5 (Adamo et al., 2004; Zhang et al., 2007) and lipopeptides to TLR2 (Firoved et al., 2004; Beaudoin et al., 2012). Activation of these TLRs on airway epithelial cells (AECs) leads to increased chemokine synthesis, such as IL-8 (CXCL8), and subsequent neutrophil recruitment to sites of infection (Greene et al., 2005; Bérubé et al., 2009; Beaudoin et al., 2013).

In addition to TLRs, the aryl hydrocarbon receptor (AhR) was recently shown to detect phenazines of *P. aeruginosa* such as pyocyanin (Moura-Alves et al., 2014). The AhR is a ligand-activated receptor/transcription factor that play important roles in adaptive responses to stress. The AhR is highly expressed by many cell types in the human lung, including epithelial cells (de Souza et al., 2014), and is the only vertebrate bHLH/PAS member that can bind and be activated by xenobiotics belonging to the polyhalogenated aromatic hydrocarbons (PHAH) class, the prototypical member of which is 2,3,7,8-tetrachlorodibenzo-p-dioxin (TCDD; dioxin). After binding its ligand, AhR translocates to the nucleus and forms a heterodimer with the AhR nuclear transporter (ARNT), leading to a AhR:ARNT complex that binds to a dioxin responsive element (DRE) to initiate gene transcription. AhR controls the transcription of hundreds of genes (Henry et al., 2010), most notably Phase I cytochrome P450 (CYP) enzymes CYP1A1, CYP1A2 and CYP1B1. AhR-dependent alterations in gene expression likely mediate the majority of dioxins' deleterious effects (Okey et al., 2005). However, there is now ample evidence to support that the AhR plays a prominent and essential role in physiological processes including the regulation of cell proliferation, apoptosis and inflammation (Thatcher et al., 2007; Baglole et al., 2008; Rico de Souza et al., 2011). A recent study also reported that the AhR protects against *P. aeruginosa* infection, as AhR-deficient mice showed increased susceptibility to infection (Moura-Alves et al., 2014).

Therefore, *P. aeruginosa* can trigger neutrophilic inflammation via at least two distinct sensor families, the TLR and AhR pathways. The relative contribution of each of these pathways to chemokine synthesis by human airway epithelial cells in response to *P. aeruginosa* is however unknown. Since neutrophilic inflammation is a key feature of CF lung disease, it is important to determine the contribution of each of these pathways to optimize intervention strategies aimed at diminishing inflammation in CF airways.

## Materials and methods

### Materials

All chemicals were from Fisher Scientific (Fair Lawn, NJ, USA). FSL-1, *S. typhimurium* flagellin and the neutralizing antibodies against Toll-like receptor 2 (TLR2) and 5 (TLR5) were bought from Invivogen (CA, USA). Pyocyanin and the AhR inhibitor CH-223191 were from Sigma-Aldrich (USA).

### *P. aeruginosa* diffusible material preparation

PA14, a highly virulent reference *P. aeruginosa* strain derived from a clinical isolate (He et al., 2004), and a PA14 mutant that does not produce any phenazines (PA14 Δ*phzS/M*) (Wang et al., 2010) graciously provided by Yun Wang were grown in LB (Luria Broth), PB (Peptone Broth, medium to maximize pyocyanin production in liquid culture) (Essar et al., 1990), or synthetic CF medium (SCFM) (Palmer et al., 2007). For planktonic cultures, PA was grown for 24 h in a shaking incubator at 37°C. For biofilm cultures, log phase PA were used to seed tissue culture wells that were left to attach for 3 h before the culture media was changed. The attached bacteria were incubated statically at 37°C for an additional 24 h. Following both growth culture methods, collected bacteria and the culture media were centrifuged and the supernatant filtered through a 0.22 μm filter.

### Epithelial cell culture

BEAS-2B airway epithelial cells were cultured as previously described (Bérubé et al., 2009). UNC CF2 airway epithelial cell lines with the most common mutation found in CF, deletion of Phe508 were kindly provided by Dr. Scott Randell (The University of North Carolina at Chapel Hill, NC, USA) (Fulcher et al., 2009). To enhance cell adherence, cells were seeded onto PureCol pre-coated plate (Advanced BioMatrix San Diego, California, USA).

### Cell lysis, RNA extraction, real-time PCR

All the techniques were performed as previously described (Bérubé et al., 2009).

### Statistical analysis

Analyses of variance (ANOVA) followed by a multiple comparison test (Bonferroni) were used to test differences in mean between groups using GraphPad Prism 6 software. *P* values < 0.05 were considered significant. ^*^p < 0.05 compared to UT cells (no inhibitor) without stimulation; ^#^*p* < 0.05 compared to UT cells (no inhibitor) under the same stimulation conditions.

## Results

### IL-8 expression in response to pyocyanin is largely dependent on AhR activation in AECs

Neutrophilic inflammation is a key pathogenic feature of Cystic Fibrosis (CF) lung disease (Cantin et al., 2015). Neutrophils are recruited to sites of infection in response to chemokines of the Cys-Xxx-Cys chemokine family, such as IL-8 (CXCL8) (Orr et al., 1980). Exposure of airway epithelial cells to *Pseudomonas*-derived products leads to the secretion of IL-8 (Bédard et al., 1993). Multiple bacterial exoproducts can lead to IL-8 synthesis, including flagellin, lipoproteins and pyocyanin (Denning et al., 1998; Zhang et al., 2007; Beaudoin et al., 2012). Whereas flagellin and lipoproteins were shown to act through TLR receptors (Zhang et al., 2007; Beaudoin et al., 2012), pyocyanin has recently been shown to act through AhR (Moura-Alves et al., 2014).

We first checked whether TLRs contributed to pyocyanin signaling and AhR to flagellin and lipoprotein signaling. In BEAS-2B AECs, TLR2, and TLR5 activation was prevented using blocking antibodies whereas the contribution of the AhR pathway was assessed using the AhR antagonist CH-223191 (Kim et al., 2006; de Souza et al., 2014).

**Figure 1:**
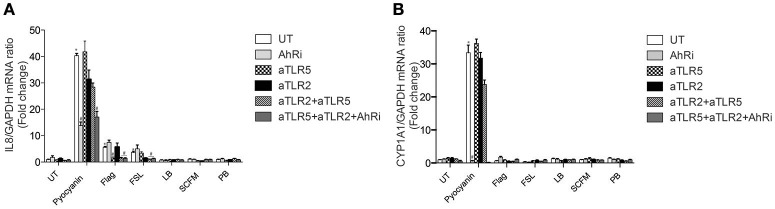
**IL-8 expression in response to pyocyanin is largely dependent on AhR in BEAS-2B AECs**. BEAS-2B AEC line were left untreated (UT) or pretreated for 1 h with 10 μM AhR inhibitor (AhRi) or/and 5 ug/ml TLR2 (aTLR2) and 5 ug/ml TLR5 (aTLR5) neutralizing antibodies. Then, cells were stimulated for 6 h with 50 μM Pyocyanin, 0.4 ug/ml flagellin, or 1 ug/ml FSL. Alternatively, BEAS-2B AECS were exposed to bacterial growth culture media LB, SCFM, or PB for 6 h. IL-8 **(A)** and CYP1A1 **(B)** gene expression were measured by qRT-PCR. LB stands for Luria Broth, PB for peptone broth and SCFM for synthetic CF medium. Results were analyzed by one-way ANOVA followed by Bonferroni post-test. ^*^p < 0.05 compared to UT cells (no inhibitor) without stimulation; ^#^*p* < 0.05 compared to UT cells (no inhibitor) under the same stimulation conditions.

TLR2 blockade prevented IL-8 expression in response to FSL-1, a TLR2/TLR6 agonist, but not TLR5 and only very slightly in response to pyocyanin (Figure 1A). Conversely, TLR5 inhibition prevented IL-8 expression in response to flagellin, its natural ligand, but not FSL-1 or pyocyanin (Figure 1A). Neither FSL-1 or flagellin induced CYP1A1 expression (Figure 1B), supporting the fact that these ligands do not act via the AhR. CYP1A1 is a well-known AhR-regulated gene (Henry et al., 2010) that serves as a reporter of AhR activity in this manuscript.

Pyocyanin-induced CYP1A1 expression was prevented by the AhR antagonist (Figure 1B). Moreover, the AhR antagonist did not affect FSL-1 or flagellin-driven IL-8 expression (Figure 1A). In contrast to FSL-1 and flagellin, pyocyanin induced both IL-8 and CYP1A1 expression. IL-8 expression was significantly decreased by the AhR antagonist, revealing that IL-8 expression is at least partially dependent on AhR activity in BEAS-2B cells exposed to the bacterial pigment pyocyanin.

### The AhR, TLR2, and TLR5 pathways contribute to IL-8 expression in BEAS-2B AECs exposed to *P. aeruginosa*

**Figure 2:**
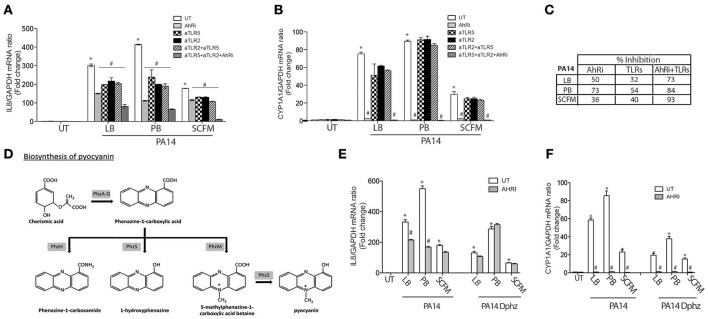
**The AhR, TLR2, and TLR5 pathways contribute to IL-8 expression in BEAS-2B AECs exposed to ***P. aeruginosa***-diffusible material**. BEAS-2B AEC were left untreated (UT) or pretreated for 1 h with 10 μM AhR inhibitor (AhRi) or/and 5 ug/ml TLR2 (aTLR2) and/or 5 ug/ml TLR5 (aTLR5) neutralizing antibodies. Then, cells were stimulated for 6 h with *P. aeruginosa* diffusible material, PA14 or PA14 Dphz (phenazine-deficient mutant of PA14), grown as planktonic cultures in luria broth (LB), peptone broth (PB) or synthetic CF media (SCFM) for 6 h. IL-8 **(A,E)** and CYP1A1 **(B,F)** gene expression were measured by qRT-PCR. Results were analyzed by one-way ANOVA followed by Bonferroni post-test. **(C)** The table represents percentage of inhibition of IL-8 by AhRi and TLRs (TLR2+TLR5). **(D)** Diagram of phenazines synthesis pathway. ^*^p < 0.05 compared to UT cells (no inhibitor) without stimulation; ^#^*p* < 0.05 compared to UT cells (no inhibitor) under the same stimulation conditions.

In CF lung disease, *P. aeruginosa* is found distally from the airway epithelium (Baltimore et al., 1989). In order to test this indirect interaction and mimic CF lung infection, we used diffusible material from *P. aeruginosa* cultures to challenge airway epithelial cells. When BEAS-2B AECs were exposed to diffusible material from the *P. aeruginosa* strain 14 (PA14) (Rahme et al., 1995) that had been grown in the common LB media, both IL-8 and CYP1A1 expression were increased (Figures 2A,B). IL-8 expression was partially inhibited by the AhR antagonist and the TLR neutralization strategy (Figure 2A). The greatest inhibition was obtained when the three pathways were blocked together, with 1/4 of the activity remaining (Figure 2C) suggesting that another unidentified pathway contribute to IL-8 expression under these conditions. CYP1A1 expression was completely dependent on the AhR pathway but unaffected by TLR inhibition (Figure 2B).

### Phenazines producing *P. aeruginosa* induces IL-8 expression in a AhR dependent manner

In the first Section (IL-8 Expression in Response to Pyocyanin is Largely Dependent on AhR Activation in AECs), it was shown that pyocyanin increases CYP1A1 and IL-8 expression in an AhR-dependent manner. Since pyocyanin production in *P. aeruginosa* is variable and influenced by the growth media, we cultured the PA14 strain in Peptone Broth (PB), a medium that enriches the production of pyocyanin and other phenazines (Essar et al., 1990) (Figure 2D). Accordingly, diffusible material prepared from bacteria grown in PB demonstrated stronger IL-8 synthesis compared to standard (LB) media (Figure 2A). This induction of IL-8 by PB-grown *P. aeruginosa* was highly dependent on the AhR pathway, resulting in 73% less IL-8 when AhR activity was inhibited by CH-223191. This percentage of inhibition was greater than the TLR pathway (54% of inhibition) (Figure 2C). CYP1A1 expression was completely dependent on the AhR pathway under these conditions (Figure 2B). Moreover, a Δ*phzS/M* mutant carrying inactivating mutations in the pyocyanin biosynthesis genes (Figure 2D) induced lower IL-8 expression that is independent of AhR (Figure 2E). Interestingly, CYP1A1 expression was significantly reduced in the Δ*phzS/M* mutant, but not abolished (Figure 2F). The remaining CYP1A1 levels were sensitive to the AhRi (Figure 2F), suggesting the presence of additional AhR ligands that do not contribute to IL-8 expression.

### Synthetic CF growth medium selects for a balanced contribution of AhR and TLR signaling to IL-8 expression

Since the bacterial growth conditions impacts the relative contribution of AhR and TLR signaling pathways in AEC IL-8 response to *P. aeruginosa*, we then tested the Synthetic CF medium (SCFM), a bacterial growth medium that mimics the nutritional composition of CF sputum (Palmer et al., 2007). In contrast to bacterial diffusible material from PB-grown *P. aeruginosa*, the SCFM-derived exoproducts led to a lesser stimulation of both IL-8 (Figure 2A) and CYP1A1 (Figure 2B). Inhibition of the AhR and TLR pathways reduced IL-8 expression to a similar extent (36 and 40%, respectively) (Figures 2A,C). Moreover, when the AhR and TLR pathways were blocked concomitantly, IL-8 was almost completely inhibited (93% inhibition) in contrast to PA14 grown in LB or PB, where the inhibition led to 73 and 84% reduction respectively (Figures 2A,C). Overall, these results show that AhR, TLR2, and TLR5 contribute to IL-8 expression, but their relative contribution is influenced by the bacterial growth condition. The AhR-dependent response is dominant in conditions that enrich for phenazine synthesis, while the synthetic CF medium elicits a balanced response between AhR and TLR-dependent responses.

### CF AECs respond similarly to BEAS-2B AECs in selecting AhR and TLR contribution to IL-8 expression

**Figure 3:**
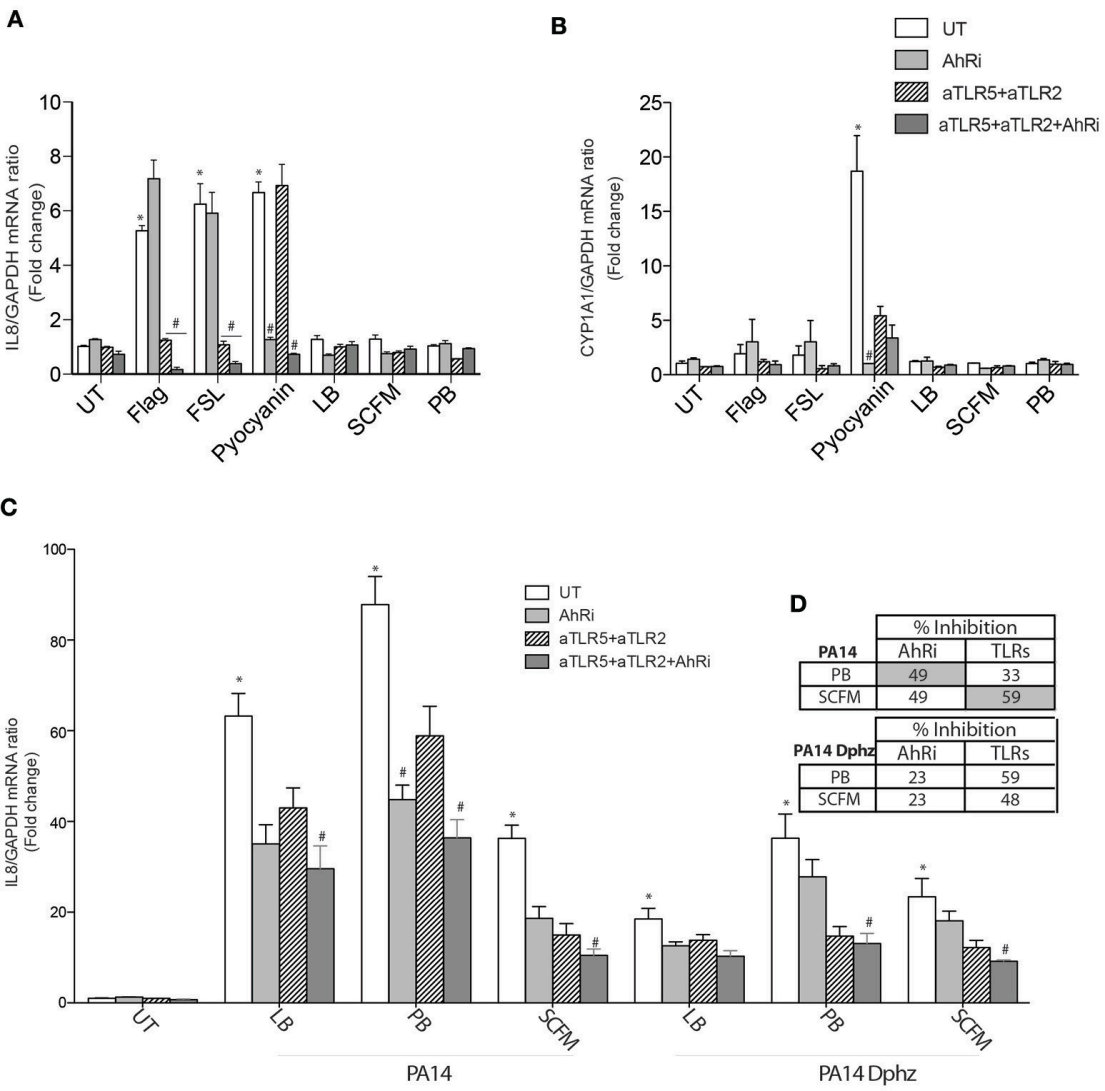
**The AhR, TLR2, and TLR5 pathways contribute to IL-8 expression in CF AECs exposed to ***P. aeruginosa-***diffusible material**. CFTRdelF508 airway epithelial cell line (CF2) were left untreated (UT) or pretreated for 1 h with 10 μM AhR inhibitor (AhRi) or/and 5 ug/ml TLR2 (aTLR2) and 5 ug/ml TLR5 (aTLR5) neutralizing antibodies. Then, cells were stimulated for 6 h with 50 μM Pyocyanin, 0.4 ug/ml Flagellin, 1 μg/ml FSL or exposed to bacterial growth culture media LB, SCFM, or PB for 6 h **(A,B)**. Alternatively CF2 AECs were stimulated with *P. aeruginosa* diffusible material PA14 or PA14 Dphz (phenazine-deficient mutant of PA14) grown as planktonic cultures in luria broth (LB), peptone broth (PB), or synthetic CF media (SCFM) for 6 h **(C)**. IL-8 **(A,C)** and CYP1A1 **(B)** gene expression were measured by qRT-PCR. Results were analyzed by one-way ANOVA followed by Bonferroni post-test. **(D)** The table represents percentage of inhibition of IL-8 by AhRi and TLRs (TLR2+TLR5). ^*^p < 0.05 compared to UT cells (no inhibitor) without stimulation; ^#^*p* < 0.05 compared to UT cells (no inhibitor) under the same stimulation conditions.

CF airway epithelial cells have an exaggerated inflammatory response (Bérubé et al., 2010; Veit et al., 2012). It is therefore essential to validate the previous results in CF AECs. Flagellin, FSL-1 and pyocyanin-induced IL-8 and CYP1A1 gene expression behaved in a similar fashion in BEAS-2B and CF AECs when exposed to TLR neutralizing antibodies and the AhR antagonist (Figures 3A,B). The relative contribution of TLR signaling was more important in CF cells exposed to bacterial diffusible material prepared from *P. aeruginosa* grown in SCFM compared to PB (59 vs. 33%) (Figures 3C,D), while AhR contribution remains essentially the same in both media (49%) (Figures 3C,D). Similarly, the Δ*phzS/M* mutant led to reduced IL-8 synthesis overall, with an even smaller contribution of AhR (23% in both PB in SCFM; Figures 3C,D).

### Biofilm diffusible material drives IL-8 expression in AECs via the AhR but not TLR signaling pathways

**Figure 4:**
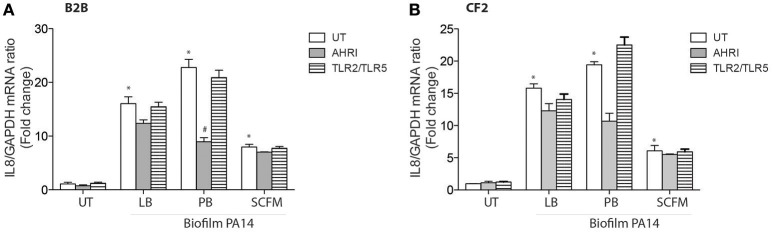
**Biofilm diffusible material drives IL-8 expression in AECs independently of TLRs with a minor contribution by AhR**. BEAS-2B AEC (Non-CF) **(A)** and CF2 (CFTRdelF508) AEC **(B)** were left untreated (UT) or pretreated for 1 h with 10 μM AhR inhibitor (AhRi) or 5 ug/ml TLR2 (aTLR2) and 5 ug/ml TLR5 (aTLR5) neutralizing antibodies. Then, cells were stimulated for 6 h with Biofilm PA14 grown in luria broth (LB), peptone broth (PB), or synthetic CF media (SCFM). IL-8 gene expression was measured by qRT-PCR **(A,B)**. Results were analyzed by one-way ANOVA followed by Bonferroni post-test. ^*^p < 0.05 compared to UT cells (no inhibitor) without stimulation; ^#^*p* < 0.05 compared to UT cells (no inhibitor) under the same stimulation conditions.

Finally, we evaluated the contribution of the AhR and TLR pathways to diffusible material prepared from biofilm instead of planktonic bacteria, as *P. aeruginosa* biofilms are characteristic of the airways of chronically infected CF patients (Kobayashi, 1995). Biofilm derived diffusible material was previously shown to stimulate AEC IL-8 expression in a TLR-independent fashion (Kravchenko et al., 2006; Beaudoin et al., 2013). Consistent with these previous observations, biofilm diffusible material prepared from PA14, whether grown in LB, PB, or SCFM increased IL-8 independent of TLRs in both BEAS-2B and CF2 AECs (Figures 4A,B). The AhR antagonist decreased IL-8 expression in PB-grown biofilm diffusible material but had only a minor or negligible contribution to LB- or SCFM-grown biofilm diffusible material (Figures 4A,B). Therefore, biofilm-diffusible material, activates a yet uncharacterized signaling pathway in the CF AEC environment with a small contribution by the AhR signaling pathway.

## Discussion

*P. aeruginosa* readily responds and adapts to its environment with changes in a wide range of protein and metabolites that are released at sites of infection and can trigger host defense responses. The host expresses a discrete set of sensors to detect these bacterial-derived ligands. Recent investigations are uncovering more sensors families of pathogens that are acting in addition to the well-characterized bacterial sensors families that comprises TLR and NOD-like Receptors (NLR). Some of these include the Bitter Taste Receptors (Lee et al., 2012) and more recently the AhR (Moura-Alves et al., 2014). These families recognize different molecular structures than those sensed by TLRs or NLRs, enabling a greater diversity in pathogen sensing. However, this raises questions regarding the contribution of these pathways to the overall inflammatory response.

In this study, we investigated the relative contribution of the well-characterized TLR family to that of the newly-discovered role of AhR as a bacterial sensor (Moura-Alves et al., 2014). This role for AhR is in addition to its previously established function as a receptor for halogenated aromatic hydrocarbons (Guenthner and Nebert, 1977). We first confirmed that pyocyanin activates the AhR in AECs (Figure 1). This was determined by evaluating CYP1A1 expression, the prototypical gene that is dependent on AhR expression and activation. Here, pyocyanin led to a significant increase in CYP1A1 expression that could be abrogated using the AhR antagonist CH-223191. Pyocyanin also led to IL-8 induction via the AhR as well as an additional pathway not involving TLR5 or TLR2. Although inhibition of TLR2 led to a small decrease in pyocyanin-driven IL-8 expression, it is most likely coming from a small contamination with lipopeptides rather than pyocyanin binding to either of the TLR2 heterodimers due to high structural selectivity of TLR2 (Zähringer et al., 2008). This is supported by the fact that no additional decrease was observed when TLR neutralization was performed in addition to inhibition of AhR. Therefore, while CYP1A1 induction by pyocyanin is entirely dependent on AhR, the IL-8 expression involves at least one other signaling pathway activated by bacterial exoproducts that have yet to be identified.

Next we determined, whether endogenous phenazines from a *P. aeruginosa* strain (PA14) grown as planktonic bacteria could trigger the same signaling pathways in airway epithelial cells (Figures 2, 3). Antagonizing AhR decreased IL-8 expression when AECs were exposed to the wildtype PA14, which correlated with a reduced IL-8 synthesis in phenazine-deficient strain, which became insensitive to AhR inhibition. Therefore, phenazines lead to IL-8 synthesis in airway epithelial cells via the AhR. It is worth noting that the phenazines-deficient PA14 strain had a reduced CYP1A1 expression but some activity remained that was sensitive to the AhR antagonist. This suggests that other AhR agonists are present that are distinct from phenazines. However, these do not appear to contribute to IL-8 synthesis since no further decrease was observed with the AhR antagonist.

Interestingly, the contribution of the various signaling pathways to IL-8 synthesis is dependent on the bacterial growth condition. When these conditions favor phenazines synthesis, AhR plays a more dominant role. While this role appears to be decreased in conditions that mimic the nutritional composition of CF sputa (Figures 2, 3), variable levels of phenazines were found in expectorated samples from CF patients that negatively correlated with lung function (Hunter et al., 2012). Therefore, both AhR and TLR pathways are playing a role in driving IL-8 synthesis in response to planktonic bacteria more typical of CF pulmonary exacerbations.

The scenario is different when biofilm growth is investigated (Figure 4). Under these conditions, IL-8 expression became independent of TLR signaling with only a minor contribution of AhR when the bacteria were grown in LB or SCFM. When the bacterial biofilms were grown in PB, the contribution of AhR was greater once again. This raises the question as to the identity of other TLR-independent pathways driving IL-8 synthesis in response to biofilm material. Interestingly, the quorum sensing molecule acyl-homoserine lactone has been shown to bind the bitter taste receptor T2R38 (Lee et al., 2012), which could be a candidate pathway that transduces biofilm-diffusible material signal into activation of inflammatory signaling in addition to AhR.

In the context of CF, where inflammation is already seen as an active participant to lung destruction, activation of the AhR by *P. aeruginosa*-derived ligands (or exposure to environmental toxicants), highlight further ways to increase destructive inflammation. This supports the importance for CF patients to avoid exposure to cigarette smoke and other similar pollutants. However, it also highlights novel ways to decrease inflammation without fully impairing host defense mechanisms. Indeed, by targeting either a single TLR or the AhR pathway while leaving others intact, it may be possible to bring back the inflammatory response within more physiological values, while retaining sufficient host defense mechanisms to avoid exposing patients to adverse risks of further infections.

The dynamic contribution of the TLR and AhR signaling pathways illustrate not only the complexity of bacterial signaling but also the possible interactions between various stressors detected by airway epithelial. Through evolution, this integration may have stemmed from different behaviors regulated by these families such as pathogen avoidance for bitter taste receptor, detoxification for the AhR pathway and expression of antimicrobial peptides by TLR required for a well-adapted response to infection.

## Author contributions

LR, SL, DN, CB, and SR have made substantial contributions to the conception, design, acquisition, analysis and interpretation of data for the work. LR and SR have drafted the work and all authors revised it critically for intellectual content. LR, SL, DN, CB, and SR have approved the final version to be published and agreed to be accountable for all aspects of the work.

## Funding

We acknowledge the financial support of Cystic Fibrosis Canada (#2595) and the Canadian Institute of Health Research (MOP#123496). The Meakins-Christie Laboratories—MUHC-RI, are supported by a Centre grant from Les Fonds de Recherche du Québec- Santé (FRQ-S). SR and CB acknowledge salary awards from the FRQ-S.

### Conflict of interest statement

The authors declare that the research was conducted in the absence of any commercial or financial relationships that could be construed as a potential conflict of interest.

## Abbreviations

AECs: airway epithelial cells
AhR: Aryl-hydrocarbon Receptor
CF: Cystic Fibrosis
CYP1A1, Cytochrome P450, family 1: member A1
IL: interleukin
PsaDM: *Pseudomonas aeruginosa* diffusible material
TLR: toll-like receptor.
